# Small-Pox in the Sheep

**Published:** 1848-01

**Authors:** 


*Small-Pox in the Sheep.*—Attention has recently been directed to the occurrence of a cutaneous disease, of an acute and fatal character, occurring among sheep. Veterinarians call the affection variola—a designation sanctioned by the authority of some distinguished members of our own profession, to whom cases of the disease have been submitted. The affection has been proved by M. Simonds,—who has given, in the *Veterinary Record*, the earliest details of its appearance—to be of a highly infectious character. Its introduction into this country has been clearly traced to infection, derived from several lots of Spanish sheep, which had been imported here and sold in the usual manner. The necessity of endeavouring to arrest the spread of so malignant a disease is a subject demanding the most active consideration. The symptoms are thus described by M. Simonds:—

"The animals in the first stage of the disease were extremely low in condition; a discharge from the nostrils, of a mucous character, was present; the breathing was quick and catching; the visible mucous membranes were inflamed, particularly the conjunctival lining of the eyelids, from which tears in increased quantities flowed; all food was refused; rumination had ceased; the ears were lopped; the head held low; and a disinclination was evinced to associate with each other, some standing and having a most dejected appearance, and others lying down. The pulse was considerably accelerated, and scarcely perceptible at the maxilla or leg, but at the heart it gave to the hand a jerking sensation; the skin was hot, red, and elevated in patches in the form of nodules, which approximated each other. The greater amount of the eruptions were on the inside of the arms and thighs and sides of the face, the labia of the female, and præputium of the male, those parts which are either nude or covered only with hair; yet, on separating the wool, the whole of the skin was seen to be similarly affected, although less intensely.

"In the second stage greater debility and emaciation existed: the discharge from the Schneiderian membrane was increased, viscid, and adherent to the alæ of the nostrils, impeding the respiration; the capillaries of the eyelids were in a highly congested state; the pulse was indistinct even at the heart; the ears and feet were cold, and the wool came off easily, showing the skin underneath to be inflamed; but the redness existed principally between the elevations; still no distinct areola was present. The summits of the nodules were blanched, arising from effusion of a very small quantity of serous fluid beneath the cuticle, which scarcely gave to it the character of a true vesicle. All the eruptions, however, had not taken on this charge.

"The third stage showed the vital power to be prostrated; the fever had become of a typhoid character, the discharge from the nostrils fœtid, and the other general symptoms much aggravated. The cuticle covering the majority of the nodules had assumed a brown colour, and pus here and there was formed on the margins of some of them, showing the ulcerative state to have commenced; in others, simple desquamation of the cuticle had begun to take place.

"In some extreme cases the ulceration had extended to the subcutaneous structure, and large unhealthy sores existed in several situations."

The treatment recommended appears to be of the ordinary routine character, and to have little influence over the progress of the disease. The importance of isolation, with the view of preventing the spread of the disease, is very properly insisted on.—*London Lancet.*

